# Facial Swelling in a Young Adult With Type 1 Diabetes: Morbihan Disease as a Scleroderma Mimic

**DOI:** 10.7759/cureus.101635

**Published:** 2026-01-15

**Authors:** Rania Shammas, Santiago Gudino-Rosales, Diana Kneiber, G. Peter Sarantopoulos, Thanda Aung

**Affiliations:** 1 Rheumatology, University of California Los Angeles David Geffen School of Medicine, Los Angeles, USA; 2 Dermatology, University of California Riverside, Riverside, USA; 3 Dermatology, University of California Los Angeles David Geffen School of Medicine, Los Angeles, USA; 4 Pathology, University of California Los Angeles David Geffen School of Medicine, Los Angeles, USA

**Keywords:** facial swelling, morbihan, rosacea, scleredema diabeticorum, scleroderma mimic

## Abstract

Scleredema diabeticorum and Morbihan disease (solid facial edema) can mimic scleroderma, creating diagnostic challenges for rheumatologists. We report a 19-year-old male with poorly controlled type 1 diabetes (glycated hemoglobin (HbA1c) >9%) presenting with progressive facial swelling. Extensive workup excluded superior vena cava syndrome and connective tissue diseases. Histopathological examination of the skin fragment was suggestive of scleredema diabeticorum or Morbihan disease secondary to rosacea. Key differentiating features included sparing of hands/feet, absence of Raynaud's phenomenon, and non-specific low-titer anti-nuclear antibody (ANA). This case emphasizes recognizing scleroderma mimics to ensure appropriate therapy.

## Introduction

Scleredema diabeticorum (Type 3 scleredema) affects 2.5-14% of diabetic patients with poor glycemic control and represents a significant diagnostic challenge in rheumatology [1]. This condition closely mimics early scleroderma through symmetric skin thickening and induration, particularly affecting the face, neck, and upper trunk [2]. The clinical overlap can lead to misdiagnosis and inappropriate immunosuppressive therapy. Morbihan disease, characterized by persistent facial edema from rosacea complications, can compound diagnostic complexity [3]. Recognition of distinguishing clinical features is crucial for appropriate management.

## Case presentation

A 19-year-old male presented to the rheumatology clinic for facial swelling and positive anti-nuclear antibody (ANA) testing. He had a history of type 1 diabetes that had been poorly controlled. About 2 years prior to presentation, he woke up with facial swelling that has been progressive since. He had some clear mucus drainage from the eyes but no fevers, sinus disease, or congestion. He initially saw his primary care physician, who gave empiric courses of azithromycin, which were ineffective. He saw an Ophthalmologist who noted a normal eye exam. He saw an allergist who performed skin allergy testing, which was normal. An angioedema panel was also normal. He was given a trial of high-dose antihistamines, which were ineffective as well. He saw a dermatologist, who gave an empiric course of doxycycline, which did not improve his symptoms. He saw an ear, nose, and throat specialist, who performed a nasal scope, which was normal. CT of the sinus was normal for sinus disease but showed nonspecific facial soft tissue swelling. He was given a 6-day course of methylprednisolone that helped temporarily but then the symptoms worsened after the course was completed. Additional lab tests were drawn showing a positive ANA test, and he was referred to rheumatology to rule out a connective tissue disease such as scleroderma.

**Figure 1:**
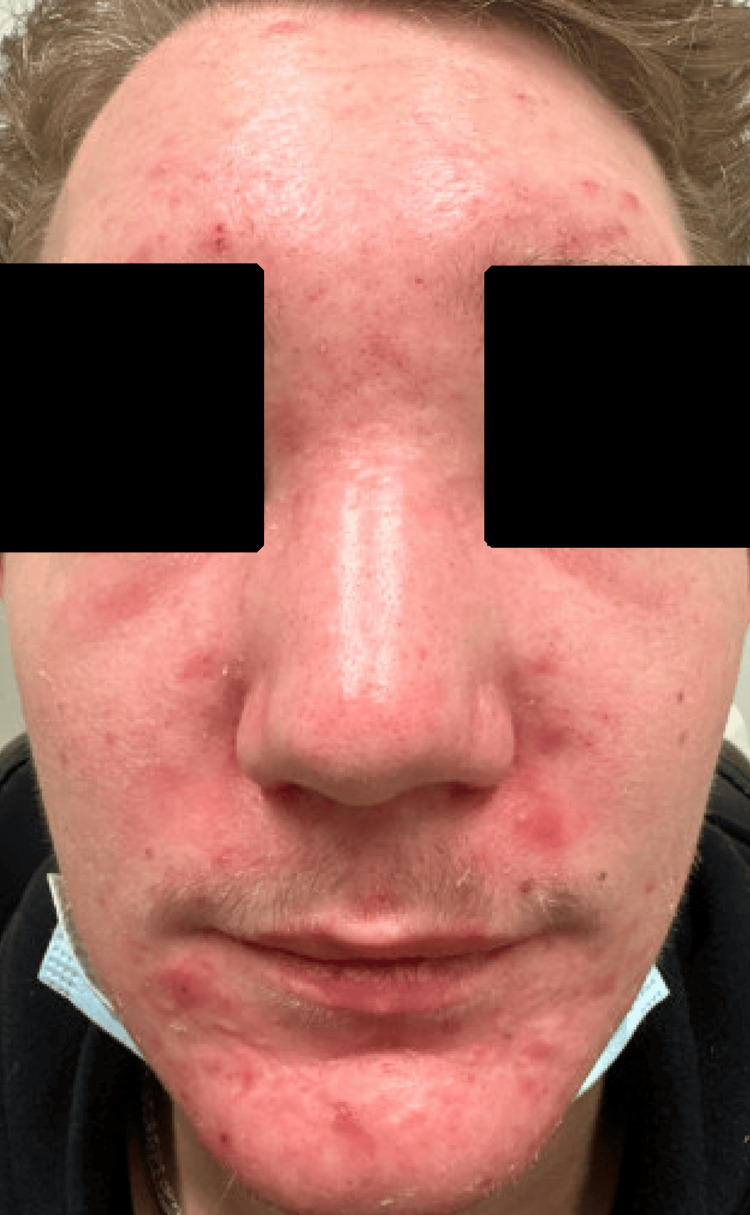
Facial swelling of the forehead and nasal bridge with scattered papules

Upon presentation, he reported progressive worsening of the facial swelling and an otherwise normal review of systems. He had no other significant past medical history besides type 1 diabetes. He was on insulin and no other medications. He did not smoke or drink alcohol, and denied any illicit drug use. He had no known family history of autoimmune disease. Physical exam was notable for facial swelling that included the forehead and nasal bridge with scattered papules (Figure 1). There were no other rashes, no peripheral edema, and no evidence of sclerodactyly, inflammatory arthritis, or Raynaud’s.

Lab results were significant for an elevated glycated hemoglobin (HbA1c) of 10.8. Complete blood counts, metabolic panel, and thyroid studies were normal. Angioedema panel and IgE levels were normal. He tested negative for hepatitis B, hepatitis C, syphilis, and HIV. ANA testing was positive at a titer of 1:80, with a speckled pattern. Inflammatory markers, which included sedimentation rate and C-reactive protein, were normal. Secondary autoimmune antibody panel was normal, including double-stranded DNA (dsDNA), SSA (or anti-SSA/Ro) antibodies, SSB (or anti-SS-B/La) antibodies, Smith, RNP (ribonucleoprotein) antibodies, antineutrophil cytoplasmic antibodies (ANCA), centromere, Scl-70, RNA polymerase, and Mi-2, Jo-1, and celiac antibodies. CT of the neck and CT chest venogram showed patent vessels with no evidence of superior vena cava syndrome.

To aid in diagnosis, a skin biopsy was performed, and the anatomopathological examination showed perivascular/periadnexal lymphoplasmacytic inflammation with mast cells and background vessel ectasia.

**Figure 2:**
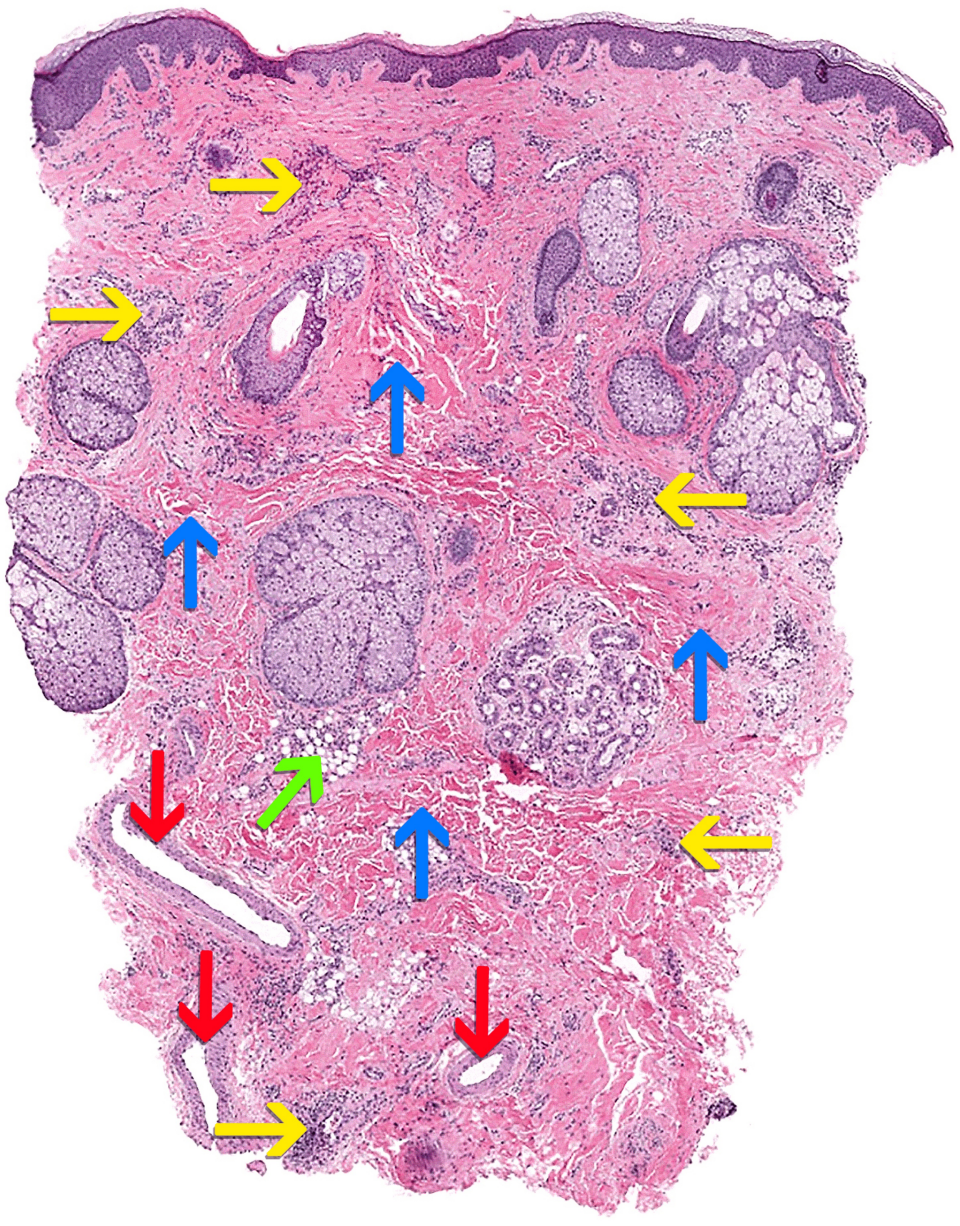
Histologic sections of skin biopsy Histologic sections show mild superficial to deep perivascular lymphoplasmacytic inflammation with scattered mast cells (yellow arrows). There is an increase in dermal and subcutaneous fibrosis (blue arrows), and diminished peri-adnexal fat (green arrow). Lymphovascular ectasia, to include dilated arterioles within deeper tissues, is seen as well (red arrows); Hematoxylin and eosin 40x.

There was also a suggestion of dermal fibrosis, characterized by decreased adipose tissue around adnexa and within the underlying subcutis. Trichrome special staining highlighted collagen, while Elastic Von Gieson special staining highlighted normal elastic fibers. Colloidal iron and alcian blue special stains highlighted mild perivascular, periadnexal, and interstitial mucin. Periodic Acid-Schiff special stain and spirochete immunohistochemistry were negative. There was also no vasculitis or malignancy. Overall, the vascular ectasia and lymphoplasmacytic inflammation with mast cells were most consistent with rosacea, and despite the lack of obvious dermal edema, Morbihan disease was in the differential, along with diabetic scleredema (Figure 2)

He was counseled on the importance of glycemic control and referred to dermatology for further help in management. Given his prior lack of response to doxycycline and systemic steroids, he was placed on isotretinoin and loratadine with some symptomatic improvement. The patient’s facial papules resolved, and his facial swelling progressively improved on therapy. Due to missing several appointments, the patient had a 1-month gap in isotretinoin therapy, which led to a rebound increase in glabellar swelling.

## Discussion

This case demonstrates the diagnostic challenges of scleredema diabeticorum and Morbihan disease (MD) as scleroderma mimics, particularly in young patients, where systemic sclerosis is more commonly considered.

Morbihan disease is a rare condition, generally considered a complication of chronic rosacea [4], though whether it represents a distinct disease remains unclear. Clinically, MD presents with symmetric, non-pitting edema and persistent erythema of the upper half of the face, involving the forehead, glabella, eyelids, and cheeks [4, 5]. While not always present, it may also be accompanied by classic features of rosacea such as telangiectasias, papules, and pustules [6]. While the exact pathogenesis is poorly understood, persistent inflammation and subsequent lymphatic obstruction are thought to contribute to its development [3, 5]. Histologically, findings are nonspecific but include dermal edema, perivascular and periadnexal inflammatory infiltrate, and dilated lymphatic vessels [3, 6-7].

Adding to the diagnostic complexity of MD, diabetic scleredema may also present with non-pitting induration of the face [8]. However, scleredema is more diffuse, involving symmetric thickening and tightening of the fingers, dorsal hands, shoulders, back, and chest, which may limit mobility [8-9]. Its pathogenesis is strongly associated with poorly controlled, longstanding diabetes mellitus [8, 10], and histology typically reveals dermal thickening with an increased accumulation of aminoglycans between collagen bundles [10-11]. Scleredema diabeticorum was first described by Cole et al. [2] as a distinct cutaneous manifestation in diabetic patients, yet it remains underrecognized as a scleroderma mimic (see Table 1 for key differentiating features).

**Table 1:** Key Differentiating Features ANA: anti-nuclear antibody

Clinical Feature	Scleroderma	Scleredema Diabeticorum	Morbihan Disease
Hand/foot involvement	Universal sclerodactyly	Characteristically spared	Spared
Raynaud's phenomenon	Present in >95%	Typically absent	Absent
Nailfold capillaroscopy	Abnormal patterns	Normal architecture	Normal
ANA specificity	Anti-Scl-70, centromere	Non-specific, low-titer	Usually negative
Organ involvement	Multisystem	Predominantly cutaneous	Cutaneous only
Associated condition	Autoimmune	Diabetes mellitus	Rosacea

The distribution pattern in our patient strongly favored MD or scleredema over scleroderma. Recent literature confirms that scleredema diabeticorum can cause significant morbidity, including restrictive lung disease in severe cases, emphasizing the importance of early recognition and glycemic optimization. The histology features suggestive of both MD and scleredema added diagnostic complexity, as both conditions can cause facial induration. However, the inflammatory component with mast cell infiltration on histology supported the rosacea-related process [6].

Management of MD is challenging, as it is often resistant to treatment and lacks standardized guidelines. Systemic isotretinoin (40 mg for 6-12 months) has reduced periocular and cheek edema in several reports, with enhanced outcomes observed when combined at lower doses (10-20 mg) with an antihistamine like ketotifen or desloratadine [3, 6]. Tetracyclines have also been used, with some patients responding to doxycycline (200 mg) or minocycline (100 mg) over 4-6 months [6]. When paired with prednisone or prednisolone (doses ranged from 10-60 mg), clinical improvement was reported, though relapses frequently occurred after steroid tapering [6].

Procedural interventions have been explored in treatment-refractory cases of MD. Blepharoplasty has been used to debulk enlarged periocular tissue and improve lymphatic drainage. One case managed with conventional blepharoplasty reported sustained results at a one-year post-operative follow-up [12], though others have documented recurrent eyelid edema after surgery [13, 14]. Used as an alternative procedure, CO2 laser blepharoplasty offered good cosmetic outcomes and improved visual function in one case [15]. With less procedural bleeding and a potentially decreased risk of recurrence, it may yield better results over standard surgical blepharoplasty [15], but further studies are needed. Lymphatic drainage massage has also been utilized with mixed results, ranging from only partial improvement of facial edema to a complete clinical resolution [16].

In addition to these interventions, a few emerging therapies have been described in isolated reports. Baricitinib, a Janus kinase (JAK) inhibitor typically used for autoimmune disorders, was trialed at 2 mg daily for four weeks in treatment-resistant MD, resulting in reduced facial erythema and morning eyelid edema [17]. In a separate case, daily oral montelukast (10 mg) combined with an intranasal cromolyn sodium spray led to a marked decrease of facial edema after three weeks of treatment, with sustained results at a one-year follow-up [18]. Although these reports are limited and long-term data remain scarce, they highlight novel options for a disease that remains challenging to manage.

Similar to MD, diabetic scleredema also presents a therapeutic challenge, with no standardized management and widely variable responses to treatment. Tight glycemic control has improved skin and joint mobility in some cases [9], though others demonstrated no cutaneous benefit [11]. Phototherapy with either ultraviolet A-1 (UV-A1) or oral photochemotherapy with psoralen plus ultraviolet A (PUVA) has shown promise, with reports of full and partial responses [11, 19]. However, non-responders have also been reported [20].

Systemic corticosteroids and immunotherapies have been explored but are typically reserved for severe disease refractory to phototherapy. With the risk of worsening diabetes, steroids are controversial, with one study showing only one of five patients achieving full resolution with them [19]. In a case of concurrent scleredema and chronic myeloid leukemia, combined treatment with corticosteroids and imatinib alternatively resulted in complete resolution of cutaneous findings [19]. This may suggest a role for immunotherapy in select cases, but further investigation is needed to clarify its efficacy and therapeutic value.

Radiation therapy (RT), though not widely studied, has revealed benefit in a few cases. Local RT softened indurated plaques and improved cervical mobility in one patient who subsequently exhibited sustained results over six years [21]. In a separate case of treatment-resistant disease, volumetric modulated arc therapy (VMAT) led to full recovery of neck and jaw mobility and complete resolution of her skin lesions three months after treatment. Furthermore, both RT and VMAT were well tolerated, respectively demonstrating only transient fatigue and dermatitis as well as localized telangiectasias [11, 22].

## Conclusions

Scleredema diabeticorum and Morbihan disease (MD) can present as scleroderma mimics that can be misleading, particularly in young patients. This case highlights essential clinical pearls: (1) mimics characteristically spare hands and feet, unlike scleroderma, (2) absence of Raynaud's phenomenon and specific autoantibodies should raise suspicion, (3) normal nailfold capillaroscopy helps exclude scleroderma, and (4) multidisciplinary care addressing concurrent conditions is essential. Although treatment is challenging for these conditions, heightened awareness of these mimics can prevent diagnostic delays and inappropriate therapy.
